# Editorial: Model-informed decision making in the preclinical stages of pharmaceutical research and development

**DOI:** 10.3389/fphar.2023.1184914

**Published:** 2023-04-12

**Authors:** Rui Li, Morgan Craig, David Z. D'Argenio, Alison Betts, Donald E. Mager, Tristan S. Maurer

**Affiliations:** ^1^ Translational Modeling and Simulation, Medicine Design, Pfizer Inc., Cambridge, MA, United States; ^2^ Department of Mathematics and Statistics, Université de Montréal, Montréal, QC, Canada; ^3^ Sainte-Justine University Hospital Research Centre, Montréal, QC, Canada; ^4^ Department of Biomedical Engineering, University of Southern California, Los Angeles, CA, United States; ^5^ Applied BioMath, LLC, Concord, MA, United States; ^6^ Department of Pharmaceutical Sciences, University at Buffalo, State University of New York, Buffalo, NY, United States; ^7^ Enhanced Pharmacodynamics, LLC, Buffalo, NY, United States

**Keywords:** drug discovery, modeling and simulation, decision-making, preclinical-to-clinical translation, preclinical stage

Although late-stage clinical attrition has been long considered as the most significant issue facing the pharmaceutical industry, the probability of technical success in the clinic is largely related to decisions made years earlier in the preclinical stages of Research and Development (R&D); it is at these early stages that decisions are made regarding the molecular target, modality of intervention, drug design and clinical candidate selection. Accordingly, model-informed drug development approaches that have proven useful in the clinic (e.g., quantitative systems pharmacology (QSP) modeling, physiologically based pharmacokinetic (PBPK) modeling, pharmacokinetic-pharmacodynamic (PKPD) modeling) are increasingly leveraged to support decisions in the earlier preclinical stages of R&D. These advances, however, have not been well-represented in the literature. This topic illustrates efforts to apply modeling in target verification, lead compound optimization, clinical candidate selection, and human efficacious dose prediction, with an emphasis on how modeling and simulation is being used to advance hypothesis driven research and support decision making in preclinical research. As a collection, the papers included in this topic will allow researchers to better understand the impact and limitations that such modeling has in real-world drug research, and, in turn, facilitate insight and guidance for future research in quantitative pharmacological modeling and simulation.

Presented as a high-level overview, authors from several pharmaceutical companies shared their collective experiences about how modeling and simulation approaches have been used to inform various decision points from discovery to first-in-human clinical trials (Kondic et al., 2022). Target validation is considered as one of the main areas where QSP can impact drug discovery, however adoption of this approach is slow due to the multiscale nature and complexity of typical QSP models (Chelliah and van der Graaf, 2022). Diving in further, Bansal et al. (Bansal et al., 2022) discuss the development of a novel QSP model to predict the drug dosing and affinity requirements for potential targets of the complement pathway. They used their model to test the feasibility of developing small- or large-molecule therapies targeting this pathway. Evaluation of the level of target engagement required for efficacy with a QSP model not only validates the feasibility of the targets, but also provides drug design teams with needed goals for identifying efficacious therapies for the feasible targets. Besides confidence in targets, successfully identifying a therapy also relies on forecasting the necessary dosing to achieve clinical efficacy. Three studies in our topic show how preclinical modeling and simulation approaches can be applied to compare and prioritize targets based on required levels of target engagement, and to identify the most promising clinical large-molecule candidates based on optimized human dosing regimens (Kapitanov et al., 2021; Dong et al., 2022; Marcantonio et al., 2022). A similar modeling strategy was also applied to predict the human efficacious dose of small-molecule NaV1.7 inhibitor (Ballard et al., 2021), and to validate a strategy to increase antibody penetration in solid tumors through transient inhibition of antibody-antigen binding (Bordeau et al., 2022).

Beyond prospective predictions, retrospective analysis of existing clinical data through PBPK modeling can provide valuable information about target engagement required for efficacy at the site of action that may not be easily assessed using experimental methods (Ayyar et al., 2022; Bloomingdale et al., 2022). These studies also help to bridge preclinical information with clinical outcome, hence facilitate future discovery and development of similar therapies. Dunlap and Cao (Dunlap and Cao, 2022) additionally discuss why careful consideration of the tissue microenvironment and physiology is critical for accurately predicting *in vivo* drug-target interactions and hence clinical outcomes.

Modeling preclinical data generated by novel tools can further help to better understand the system, facilitate applications of these tools in drug discovery, and provide the foundation for preclinical-to-clinical translation (Parra-Guillen et al., 2021; Lewin et al., 2022). Computational methods, including machine learning, are increasingly used in early drug discovery. A novel computational method to predict the synergistic effects of drug combinations is included in this topic (Nafshi and Lezon, 2021). More recently, Brubaker et al. (Brubaker et al., 2019) developed a method to computationally translate genomic responses to bridge the gaps between lab animals and human. This approach shows good promise for pushing the field of model-informed drug development forward, as translational modeling work is typically based on phenotypic data.

In conclusion, this topic highlights exciting new approaches to advance preclinical drug development and help reduce attrition along the drug development pipeline.
